# Changes in contrast sensitivity after surgery for intermittent exotropia

**DOI:** 10.1038/s41598-022-10399-2

**Published:** 2022-04-21

**Authors:** Young Hwan Bae, Dong Gyu Choi

**Affiliations:** Department of Ophthalmology, Kangnam Sacred Heart Hospital, Hallym University College of Medicine, 665 Shiheongdae-ro, Seoul, 07442 South Korea

**Keywords:** Diseases, Medical research

## Abstract

To determine whether intermittent exotropia (IXT) surgery affects contrast sensitivity (CS), this retrospective study evaluated the changes in monocular and binocular CS and the binocular summation ratio (BSR) quantified as the ratio between the values of the binocular and the better monocular CS score (BSR = binocular CS score/better monocular CS score) after surgery for IXT. The subjects were patients who had undergone IXT-correcting surgery with a postoperative follow-up of > 3 months and had pre- and postoperative records of Mars CS test scores. In total, 64 patients (128 eyes) were evaluated. Both the binocular and monocular CS scores of the operated eyes were significantly worse on postoperative 1 day than the preoperative scores, but they were recovered after 1 week. The monocular CS scores of the operated eyes were significantly worse than those of the non-operated eyes until 1 week. There was no significant difference in monocular CS scores between the one-muscle and two-muscle surgeries and in binocular CS scores between the successful alignment and overcorrection groups even on the first day after surgery. The mean BSR was significantly decreased until postoperative month 1, however, recovered to preoperative levels after month 3. In conclusion, IXT-correcting surgery may temporarily worsen the CS, but it is recovered to preoperative levels. Thus, changes in CS in the immediate postoperative period after strabismus surgery should not be of concern.

## Introduction

Visual performance is mainly measured by letter charts, such as the Snellen chart that uses high-contrast black letters on a white background. However, some individuals with good visual acuity (VA) in the Snellen chart test at a very high contrast may be visually handicapped in real-life situations. Therefore, measuring other factors, including contrast sensitivity (CS), color vision, or glare, may be helpful for a more accurate assessment of the quality of vision^1–3^. The CS test represents the spatial resolution of the visual system, which can be abnormal in many diseases including amblyopia, optic neuritis, cataract, glaucoma, strabismus, and brain lesion^4,5^. Several studies have investigated CS in exotropia patients under different conditions, such as the presence or absence of glare and degree of illumination^6–9^. However, to date, only a limited number of studies have evaluated the changes in monocular and binocular CS according to surgical outcomes after strabismus surgery.

Therefore, this study aimed to determine whether intermittent exotropia (IXT) surgery affects CS scores. We investigated the changes in the monocular and binocular CS, and the binocular summation, which was defined as the superiority of the binocular over monocular performance after surgery for IXT^10^. In addition, the CS scores were analyzed according to the surgical outcomes (successful alignment versus overcorrection) and surgical methods for correcting IXT.

## Methods

### Study design and subjects

This was a retrospective study of patients who had undergone surgery for IXT with postoperative follow-up of > 3 months and had records of pre- and postoperative monocular and binocular CS scores. The exclusion criteria were as follows: (1) history of eye operations including ocular surgery; (2) restrictive, paralytic, or sensory exotropia; (3) other ocular diseases; or (4) systemic abnormalities such as nervous system disease or developmental delay. Patients with A or V pattern, oblique muscle dysfunction, or dissociated vertical deviation not requiring surgical correction were not excluded.

The study protocol adhered to the Declaration of Helsinki and was approved by the Institutional Review Board of Hallym University Kangnam Sacred Heart Hospital (approval no. 2020-11-012). The need for informed consent was waived owing to the retrospective nature of the study by the Institutional Review Board of Hallym University Kangnam Sacred Heart Hospital.

### Preoperative evaluation

The following preoperative characteristics were reviewed: age at surgery, sex, best-corrected VA, pre- and postoperative deviations at distance and near, presence of amblyopia, associated strabismus such as dissociated vertical deviation, vertical deviation, and oblique muscle dysfunction and sensory status assessed using the Titmus stereotest (Stereo Optical Co., Inc., Chicago, IL, USA) and Worth-4-Dot test (Richmond Products, Albuquerque, NM, USA).

All patients underwent a complete ocular examination preoperatively. Cycloplegic refraction was performed after instilling 1% tropicamide (Mydriacyl, Alcon Lab. Inc., Fort Worth, TX, USA) and 1% cyclopentolate hydrochloride (Cyclogyl, Alcon Lab. Inc.). The angle of deviation was measured using the alternate prism cover test with accommodative targets both at distant (5 m) and near (33 cm) with their glasses. Amblyopia was defined as the VA difference of two lines or more between the two eyes. Vertical deviation was defined as hypertropia/hypotropia of more than 5 prism diopters (PD) in the primary position. Sensory status was assessed in all subjects. When a patient saw four dots during the Worth four-dot test at distance, the result was recorded as “fusion”, and stereoacuity of 100 s of arc or better using the Titmus stereotest at near was defined as “good stereopsis” .

### Measurement of CS scores and quantification of the binocular summation ratio (BSR)

The CS was assessed using the Mars contrast sensitivity test (The Mars Perceptrix Corporation, Chappaqua, NY, USA). Briefly, the Mars test is used to measure spatial CS at peak sensitivity. It is a handheld chart with a sequence of 48 randomly ordered 10 different letters. Measurements to the right eye, left eye, and both eyes are performed individually with three separate charts with the best optical corrections. Each chart has eight lines with six letters per line with a letter size of 6/145 Snellen, which is approximately 0.8 cycles per degree. In this study, the Mars test was performed by one of three trained technicians with a luminance of approximately 90 cd/m^2^ and 50 cm from the patient. The logarithmic value was used for the analysis of CS, which was defined as “CS scores”. Patients were encouraged to get the correct answer with each correct letter representing an accumulation of 0.04 log units. The test ended when two consecutive letter-reading errors were made, and the final score was the log CS of the last successful letter, with a decrement of 0.04, for any wrong answers that precede the two continuous errors^11,12^.

Using CS scores, the BSR was quantified as the ratio between the values of the binocular and the better monocular CS score. (BSR = binocular CS score/better monocular CS score).

### Strabismus surgery and postoperative management

Bilateral lateral rectus muscle recession (BLR) or unilateral lateral rectus recession-medial rectus resection (R&R) was performed under general anesthesia by a single surgeon (DGC) who had no preference for either procedure. Unilateral lateral rectus recession (ULR) was commonly performed for exotropia of $\le \hspace{0.17em}$25 PD at distance and near. The surgical dosage was based on the distant angle of exodeviation, as shown in Table 1. The surgical methods were decided by the surgeon; however, surgeries were performed after discussion with the patients or their parents about one-eye or two-eye surgery and one-muscle or two-muscle surgery in 20–25 PD exotropia.

**Table 1:** Surgical dosage for intermittent exotropia.

PD	BLR (mm)	R&R (mm)	ULR (mm)
15	4.0	4.0/3.0	8.0
20	5.0	5.0/4.0	9.0
25	6.0	6.0/5.0	10.0
30	7.0	7.0/5.5	
35	7.5	7.5/6.0	
40	8.0	8.0/6.5	
50	9.0	9.0/7.0	

*PD* prism diopters, *BLR* bilateral lateral rectus recession, *R&R* unilateral lateral rectus recession-medical rectus resection, *ULR* unilateral lateral rectus recession.

Eye alignment at distance and near fixation in the primary position was measured at 1 day, 1 week, 1 month, and 3 months postoperatively. Patients who had esodeviation or diplopia postoperatively were prescribed alternate full-time patching until the disappearance of diplopia or esodeviation. If esotropia or diplopia persisted by 2 months, cycloplegic refraction was rechecked, the residual or recurrent hyperopia was corrected, and base-out Fresnel press-on prisms (3M Press-On OpticsTM; 3M Health Care, St Paul, MN, USA) were prescribed until the esotropia disappeared.

### Outcome measures

The main outcome measures were (1) the postoperative changes in BSR, monocular and binocular CS scores in the overall population, (2) the difference in the monocular CS scores between the operated and non-operated eyes, (3) the difference in the monocular CS scores of operated eyes between the eyes with one-muscle surgery (unilateral rectus recession) and two-muscle surgery (R&R), (4) the difference in binocular CS scores between the successful alignment group (defined as an alignment between 10 PD exodeviation and 5 PD esodeviation at distance and near) and overcorrection group (defined as > 5 PD esodeviation at distance and near) (5) the correlations of the binocular CS score and BSR with clinical characteristics, such as deviation angles at distance and near and sensory status measured by the Titmus Stereotest and Worth-4-dot test at month 3. The possible preoperative factors affecting CS scores were also investigated.

### Statistical analysis

The Mann–Whitney *U* test, Spearman correlation test, and Kruskal–Wallis test were used to analyze the association between preoperative demographic data and CS scores. A paired *t* test was used to compare between the preoperative and postoperative CS scores and BSR. The Mann–Whitney *U* test and Spearman correlation test were used to analyze the correlation of the postoperative binocular CS score and BSR with the postoperative sensory status and exodeviation. An independent samples *t *test was used to analyze monocular CS scores according to surgical methods and binocular CS scores according to the postoperative deviation. All statistical analyses were performed using SPSS software, version 25 (SPSS Inc., Chicago, IL, USA). A P value of < 0.05 was considered statistically significant.

## Results

In total, 64 patients (128 eyes) were included in the study. The clinicodemographic patient characteristics and their statistical correlations with the preoperative CS scores are presented in Table 2. There were 35 men and 29 women, and the mean age at surgery was 12.18 ± 9.73 years (range, 2.33–64.42). The mean preoperative VA in logMAR was 0.04 ± 0.07 (range, 0–0.40), and the mean spherical equivalent was − 1.69 ± 2.22D (range, − 11.0 to + 1.88D). The mean preoperative angle of deviation was 24.53 ± 7.61 PD (range, 15–50 PD) at distance and 28.26 ± 9.43 PD (range, 6–55 PD) at near. Good stereopsis (≤ 100 s of arc) in Titmus stereotest was achieved in 71.9%, and fusion in the Worth-4-dot test was achieved in 37.5%. Of the 64 patients, R&R was performed in 47 patients, ULR in 14 patients, and BLR in 3 patients.

**Table 2:** Clinicodemographic patient characteristics (n = 64 patients, 128 eyes) and their statistical correlation with preoperative CS scores.

	Total (n = 64)	P value
Sex (male:female)	35:29	0.345*
Age at surgery (years)	12.18 ± 9.73 (2.33–64.42)	0.689^†^
Preoperative visual acuity (logMAR)	0.04 ± 0.07 (0–0.40)	0.709^†^
Preoperative spherical equivalent (Diopters)	− 1.69 ± 2.22 (− 11.0 to 1.88)	0.089^†^
**Preoperative exodeviation (PD)**		
At distance	24.53 ± 7.61 (15–50)	0.533^†^
At near	28.26 ± 9.43 (6–55)	0.549^†^
Amblyopia, n (%)	9 (14.1)	0.617*
Dissociated vertical deviation, n (%)	2 (3.1)	0.223*
Vertical deviation, n (%)	16 (25.0)	0.705*
Oblique muscle dysfunction, n (%)	8 (12.5)	0.878*
**Sensory status**		
Good stereopsis, n (%) (100 arcsec or better in Titmus test)	46 (71.9)	0.032*
Fusion on Worth-4-dot test, n (%)	24 (37.5)	0.248*
**Surgical procedures, n (%)**		0.281^‡^
R&R	47 (73.4)	
ULR	14 (21.9)	
BLR	3 (4.7)	

*PD* prism diopters, *BLR* bilateral lateral rectus muscle recessions, *R&R* unilateral lateral rectus recess-medical rectus resection, *ULR* unilateral lateral rectus recession.

Vertical deviation = 5 PD or more hypertropia/hypotropia in the primary position.

*Mann–Whitney *U* test, ^†^Spearman correlation test, ^‡^Kruskal–Wallis test.

Significant values are in bold.

Among the preoperative characteristics, stereopsis measured by Titmus stereotest was the only factor significantly associated with the CS score (P = 0.032, Mann–Whitney *U* test). Patients with good stereopsis (defined as 100 arcsec or better in Titmus test) had significantly better binocular CS scores. The mean preoperative BSR was 1.015 ± 0.030, which significantly decreased at postoperative day 1 (1.006 ± 0.028) and month 1 (1.006 ± 0.025) (P < 0.05); however, it recovered to preoperative levels after month 3 (1.016 ± 0.022). The mean binocular CS score (logarithmic scales) was significantly lower at postoperative day 1 than at the preoperative period (1.72 ± 0.05 vs 1.75 ± 0.04, P < 0.001). However, it was recovered to preoperative levels after 1 week (P > 0.05). Similarly, the monocular CS score of the operated eyes was also significantly lower at postoperative day 1 than at the preoperative period (1.65 ± 0.81 vs 1.71 ± 0.06, P < 0.001) and was recovered to preoperative levels after 1 week (P > 0.05). Meanwhile, there was no significant change in the mean monocular CS score of the non-operated eyes (P > 0.05) (Table 3).

**Table 3:** Comparison between pre- and postoperative mean binocular and monocular CS scores.

	Binocular CS scores (n = 64)	Monocular CS scores	
		Operated eyes (n = 71)	Non-operated eyes (n = 65)
Preoperative	1.75 ± 0.04	1.71 ± 0.06	1.71 ± 0.06
**Postoperative**			
1 day	1.72 ± 0.05*	1.65 ± 0.81*	1.71 ± 0.07
1 week	1.78 ± 0.43	1.70 ± 0.07	1.72 ± 0.06
1 month	1.74 ± 0.49	1.71 ± 0.07	1.72 ± 0.08
3 months	1.76 ± 0.43	1.72 ± 0.06	1.72 ± 0.05

*P < 0.05, significant changes compared to preoperative values (paired *t* test).

Significant values are in bold.

**Figure 1:**
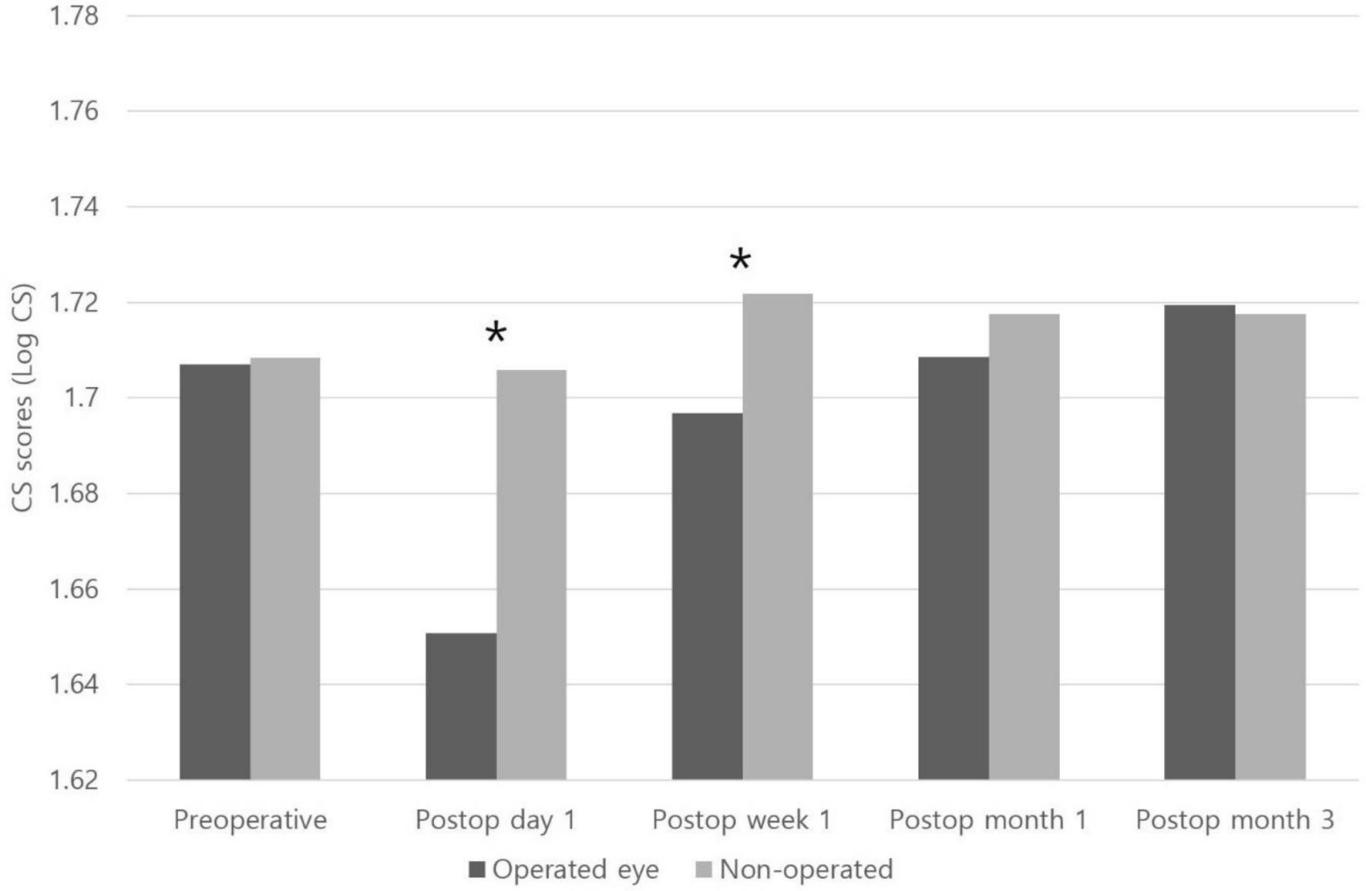
Comparison of the monocular CS scores between operated eyes (n = 71) and non-operated eyes (n = 65). *Statistically significant difference between the 2 groups using the independent samples *t* test (P < 0.05).

**Figure 2:**
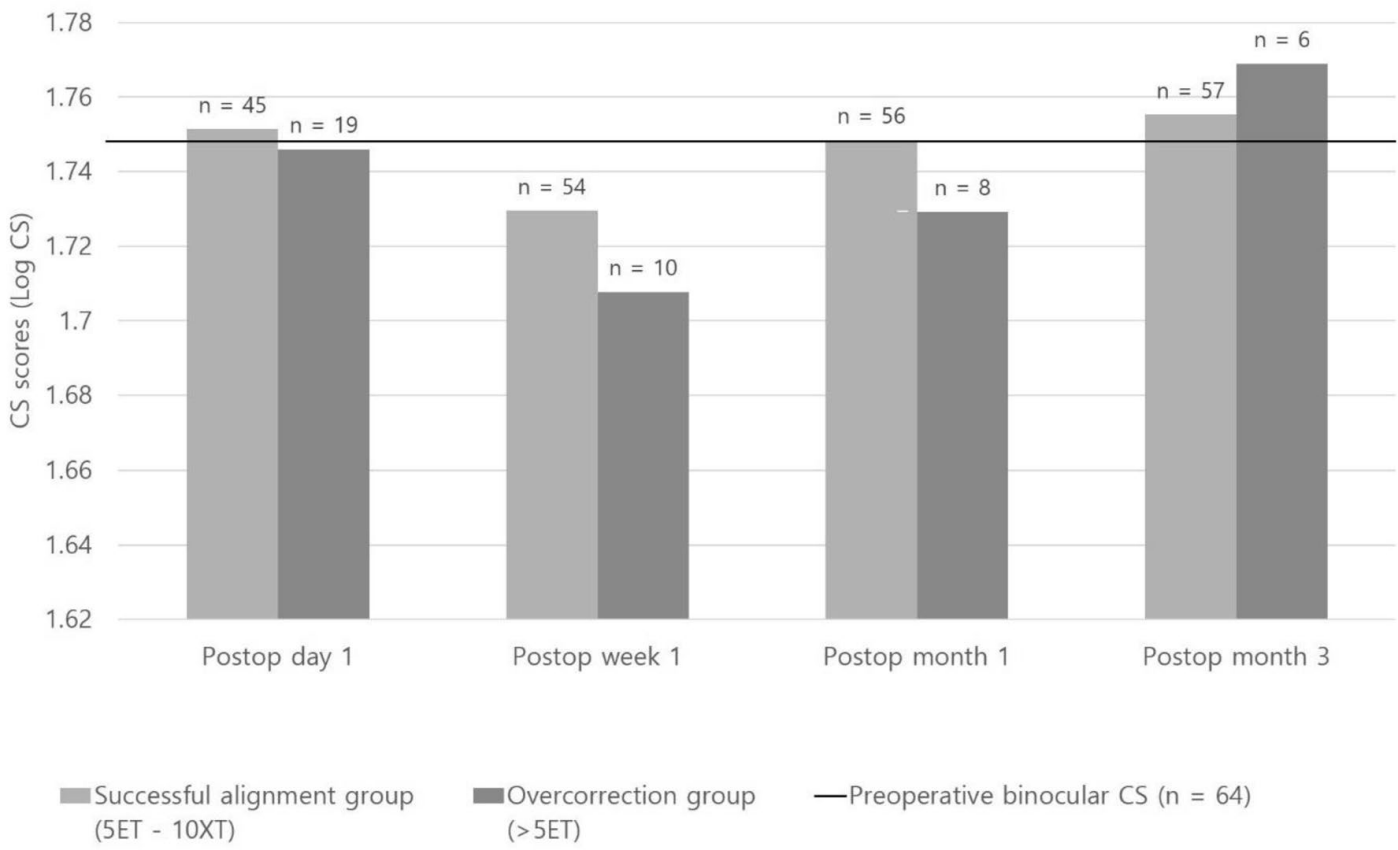
Comparison of the binocular CS scores between the successful alignment group and the overcorrection group.

**Figure 3:**
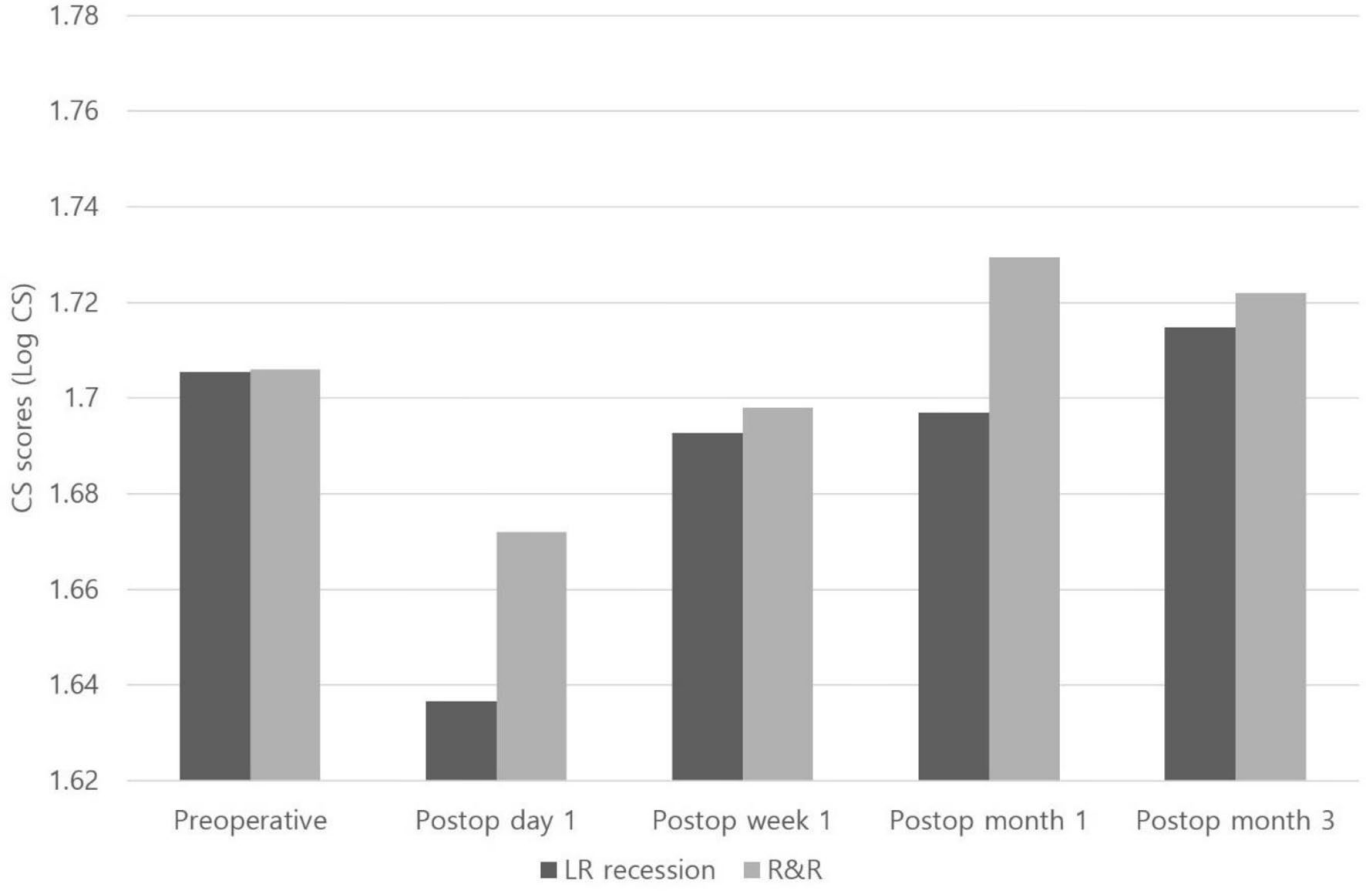
Comparison of the monocular CS scores in operated eyes between one-muscle surgery (lateral rectus recession, n = 14 eyes) and two-muscle surgery (R&R, n = 47 eyes).

The mean monocular CS score of the operated eyes was significantly worse than that of non-operated eyes at postoperative day 1 and week 1; however, the difference was no longer significant starting from 1 month postoperatively (Fig. 1). There was no significant difference in binocular CS scores between the successful alignment group and overcorrection group on every visit (Fig. 2, P > 0.05). Further, there was also no significant difference in monocular CS scores from the first day after surgery to the last visit between the eyes with one-muscle surgery (unilateral rectus recession) (n = 14 eyes) and two-muscle surgery (R&R) (n = 47 eyes) (Fig. 3, P > 0.05).

In the correlation analysis of the binocular CS score with the clinical characteristics at postoperative month 3, the mean binocular CS score was significantly higher in patients with good stereopsis (100 arcsec of better) (P < 0.05); however, it did not correlate with the exodeviation angles (P > 0.05). In the correlation analysis of the BSR with the deviation angles and sensory status, no correlation was observed (Table 4). Meanwhile, preoperatively, the proportions “good stereopsis” on the Titmus test and “Fusion” on Worth-4-dot test were 89.06% and 37.50%, respectively, which temporarily decreased at postoperative day 1 to 59.38% and 17.19%, respectively, and gradually recovered to the preoperative level until 3 months (90.64% and 37.50%).

**Table 4:** Correlation binocular contrast sensitivity (CS) score and binocular summation ratio with sensory status and deviation angles at postoperative month 3.

	Sensory status		Deviation angles (PD)	
	Good stereopsis (100 arcsec or better)	Fusion on Worth-4-dot test	At distance	At near
Binocular CS score	P = 0.027*	P = 0.810*	P = 0.964^†^	P = 0.275^†^
Binocular summation ratio	P = 0.629*	P = 0.190*	P = 0.756^†^	P = 0.727^†^

*PD* prism diopters.

*Mann–Whitney *U* test, ^†^Spearman correlation test.

Significant values are in bold.

## Discussion

CS refers to the visual ability to detect differences in luminance between an object and its background^13,14^. Several instruments, including the Contrast Glare tester 2000 (CGT-2000; Takagi Seiko, Nagano, Japan), Optec 6500 vision testing system (Stereo Optical Co., Inc., Chicago, IL, USA), and CSV 1000 (Vector Vision, Dayton OH), have been recently used to measure CS under different spatial frequencies, luminance, and presence or absence of glare^6–9,15,16^. However, their clinical use is restricted because of their limited availability and the time-consuming measurements. In this study, we used the widely used Mars contrast sensitivity test, which has good validity and reasonable agreement with the Pelli–Robson chart^11,17,18^. The Mars CS test can be used relatively easily and quickly in clinical practice, and its clinical results have been published in several studies^15,19,20^. Current guidelines in the Mars Letter Contrast Sensitivity Test USER MANUAL (http://www.marsperceptrix.com) suggest that 1.80 logCS units and 1.68 logCS units are normal results for young patients assessed monocularly and for patients aged over 60 years, respectively. Other studies reported slightly different values, with mean normal results of 1.74 logCS units or 1.62 logCS units across age groups^17,18^. In this study, the mean preoperative binocular CS score was 1.75 ± 0.04, and the mean monocular CS score of the operated eyes and non-operated eyes was 1.71 ± 0.06. These results are similar to those of previous reports^17,18^, although all subjects had intermittent exotropia.

Kim et al. reported an improvement in binocular CS scores under photopic conditions at almost all visual angles after surgery for IXT^7^. However, in our study, the binocular CS score decreased at postoperative day 1 but then recovered to the preoperative level after 1 week.

Kwon et al. reported that a poorer degree of stereopsis was associated with lower CS, and better fusional ability was associated with higher CS under mesopic conditions^8^. Therefore, the CS test is helpful for evaluating sensory function in IXT. Our study showed similar results; patients with good stereopsis (defined as 100 arcsec or better on the Titmus test) had significantly better binocular CS scores. At the postoperative month 3, the binocular CS scores and proportion of patients with ‘good stereopsis’ and fusion on Worth-4-dot recovered to the preoperative levels and patients with good stereopsis obtained statistically higher binocular CS scores, which suggest that the change in the sensory status, especially stereopsis, could have an effect on the changes in the postoperative CS scores.

In the correlation analysis for preoperative monocular CS scores of 128 eyes, distance VA was not statistically correlated with the CS scores. Considering the test distance of Mars contrast sensitivity test (50 cm), the analysis should be performed with the near VA. However, we routinely checked the Snellen VA at 5 m at every visit, and the correlation of the CS scores was analyzed with distant VA because of the retrospective nature of this study. Several studies have reported that CS worsened after ocular surgeries, such as laser in situ keratomileusis and macula-on rhegmatogenous retinal detachment, which might be due to decreased central VA after surgery^16,21,22^. However, even patients with good postoperative VA have decreased CS, and this could possibly be due to factors other than VA such as surgical stress and visual discomfort^23^. CS is temporarily decreased after refractive surgeries but is recovered after 3 months^16^.

The corneal stromal remodeling and wound healing process after refractive surgeries would contribute to diffusing light rays passing through the cornea, resulting in decreased subjective measures of CS^16^. In our study, the mean binocular CS score and the monocular CS score of the operated eyes were significantly decreased at postoperative day 1. However, there was no significant difference between the pre- and post-operative values of CS in non-operated eyes. The monocular CS score of the operated eyes was significantly worse than that of the non-operated eyes until 1 week, but it returned to the baseline level after 1 month. CS score recovery to preoperative levels was faster in IXT surgery than in refractive surgery. Moreover, there was no significant difference in monocular CS scores between the eyes with one-muscle surgery and those with two-muscle surgery, even on the first day after surgery. Therefore, the worsening of CS scores during the short-term postoperative period may have been caused by other factors instead of the corneal structural change in cases of refractive surgeries. These factors include surgical stress or difficulty in opening the eyes due to foreign body sensation or epiphora caused by sutured knots, discomfort in conjunctival incision sites, and chemosis, which would have occurred temporarily after surgery although the test was performed after irrigation if there was some discharge interrupting the measurement^24,25^. This study had some limitations. First, as it was a retrospective study, and the reproducibility and repeatability of the CS scores were not assessed. Second, our study included both children and older adult subjects, leading to some bias in the interpretation of the results of the CS scores. However, the statistical analysis using the Spearman correlation test showed that the preoperative CS scores were not affected by age at surgery (P = 0.389). Finally, a comparison with normal controls could not be performed. Further prospective clinical studies are needed to evaluate the reproducibility and repeatability of the test with a larger number of subjects. However, this study is meaningful in that it measured and analyzed monocular and binocular CS four times after IXT surgery using Mars test which is repeatable, is low cost and time efficient, and a standardized scoring method for assessing CS.

In conclusion, surgery for intermittent exotropia does not affect CS measurement in the long-term. Thus, changes in CS in the immediate postoperative period after strabismus surgery should not be of concern.

## Data Availability

The datasets generated during and/or analysed during the current study are available from the corresponding author on reasonable request.
